# Supplementary material for: Chromothripsis during telomere crisis is independent of NHEJ, and consistent with a replicative origin
Source: Genome Res. 2019 May;29(5):737–49. doi: 10.1101/gr.240705.118 (PMC6499312; doi:10.1101/gr.240705.118)
Supplement: Supplemental Material [file supp_gr.240705.118_Supplemental_file_1.zip › contigs/annotated_contigs/DB57/contig.2.DB57_length_372_mean_cov_2.82258064516.docx]

**DB57_length_372_mean_cov_2.82258064516**

```
GAGCTCAGCCAGCTGATGAGTAGGC|AGCT|GCTCTAAGGTGTAACAATAATTACTTTCCTCCAAAATGTACAATGTTCCTCTTGCGTA
 >chr15:100877088-100877117 - E=6e-06 >chr15:98690145-98690492 + E=5e-189
TTTAATTGACTTTGGCAATCCATATTTAAGACAATGAGGGGAGTTGTCAGCAATGTGAAGACAGAAATGAAGAGAAAGCAAATCGCCGT

CATTGGTATCTAAAACGTGCTGCGTATTTTAACTCCTGGGAATATTTTCTCTGGTTGGTCTTCATCTTGGAGCAATCTGCCTGATGAAA

TTTTTCTGGCCCACTGTGTTTTCTTTCTTTTGGGCCTTTCTCCTCTTGATTTTTTCTTCAAGGGCTCTCTGGTAAATGGCAGGACCCAG

TGTTCAAGTCAGCACTTC
```
